# Supplementary material for: Extending the range of graph neural networks with global encodings
Source: Nat Commun. 2026 Feb 18;17:1855. doi: 10.1038/s41467-026-69715-3 (PMC12920779; doi:10.1038/s41467-026-69715-3)
Supplement: Supplementary file 1 — Supplementary Information [file 41467_2026_69715_MOESM1_ESM.pdf]

Supplementary Information

Extending the Range of Graph Neural Networks with Global Encodings

Alessandro Caruso[1†], Jacopo Venturin[1,2†], Lorenzo Giambagli[1], Edoardo Rolando[1], Zakariya El-Machachi[1], Frank Noè[1,2,3,4*], Cecilia Clementi[1,4,5*]

[1]Department of Physics, Freie Universität Berlin, Arnimallee 12, Berlin, 14195, Germany.
[2]Department of Mathematics and Computer Science, Freie Universität Berlin, Arnimallee 12, Berlin, 14195, Germany.
[3]AI4Science, Microsoft Research, Karl-Liebknecht Str. 32, Berlin, 10178, Germany.
[4]Department of Chemistry, Rice University, 6100 Main Street, Houston, 77005, Texas, USA.
[5]Center for Theoretical Biological Physics, Rice University, 6100 Main Street, Houston, 77005, Texas, USA.

*Corresponding author(s). E-mail(s): frank.noe@fu-berlin.de; cecilia.clementi@fu-berlin.de;
†These authors contributed equally to this work.

Supplementary Note 1: The RANGE architecture

As illustrated in Fig. 1 of the main text, the RANGE architecture combines a local message-passing with an aggregation of all the network nodes into a master node M, followed by a broadcasting that redistributes the collected information back into the single nodes, effectively realizing long-range message-passing. The details on the aggregation and broadcast phases are provided below.

1.1 Aggregation

Since a multi-head attention system is implemented, master nodes funnel information into L d-dimensional spaces: the information stored in each subspace is concatenated into a h-dimensional vector so that $Ld = h'$. The aggregated embedding is

$$\mathbf{H}^{(t)} = \sigma\left(\|_{l=1}^{L} \sum_i \hat{\alpha}_i^l A_V^l \tilde{\mathbf{h}}_i^{(t)} \right), \tag{1}$$

where $\mathbf{H}^{(t)} \in \mathbb{R}^{h'}$ is the embedding of M, σ is an element-wise non-linear activation, $\|_{l=1}^{L}$ represents the concatenation operator, $A_V^l : \mathbb{R}^h \to \mathbb{R}^d$ is a learnable matrix, and $\tilde{\mathbf{h}}_i^{(t)}$ refers to the i-th node embedding after a local message-passing iteration. Based on the conventional implementation of additive self-attention [1, 2], the weight $\hat{\alpha}_i^l$ of embedding i and head l is defined as:

$$\alpha_i^l = (\mathbf{a}^l)^\top \text{LeakyReLU}(A_Q^l \mathbf{H}^{(t-1)} + A_K^l \tilde{\mathbf{h}}_i^{(t)} + A_E^l \mathbf{E}_i) \tag{2}$$

$$\hat{\alpha}_i^l = \text{Softmax}(\alpha_i^l) = \frac{\exp \alpha_i^l}{\sum_j \exp \alpha_j^l}. \tag{3}$$

Here, $A_Q^l : \mathbb{R}^{h'} \to \mathbb{R}^d$, $A_K^l : \mathbb{R}^h \to \mathbb{R}^d$ and $A_E^l : \mathbb{R}^f \to \mathbb{R}^d$ are learnable matrices and $\mathbf{a}^l \in \mathbb{R}^d$ is a learnable vector. The query projection matrices A_Q^l always act on the previous virtual node embedding $\mathbf{H}^{(t-1)}$. The edge features between master node and the graph nodes, denoted as a function of their respective distances $\mathbf{E}_i = \text{RBF}(r_i)$, are carefully designed to extend the standard radial basis expansion and accommodate non-bounded distances without introducing a cutoff. We achieve this by scaling the distances between M and the graph nodes by their maximum

$$r_i = \frac{||\mathbf{x}_i - \mathbf{X}_M||}{\max_j ||\mathbf{x}_j - \mathbf{X}_M||} \quad \in [0, 1], \tag{4}$$

where $\mathbf{x}_i$ denotes the position of node i and $\mathbf{X}_M$ is the position of the master node, $\frac{1}{N}\sum_i \mathbf{x}_i$. The new distances are then transformed into edge features via radial basis expansion. This allows for complete transferability of the trained network across different system sizes.

1.2 Broadcast

In order to update the embeddings of the base graph with the aggregated information while retaining learned short-range interactions, we apply self-loops to each atomic node in the attention mechanism as follows:

$$\mathbf{h}_i^{(t+1)} = \text{MLP}\left(\|_{l=1}^{L} \left(\hat{\beta}_{i,\text{self}}^l B_{V,\text{self}}^l \tilde{\mathbf{h}}_i^{(t)} + \hat{\beta}_i^l B_V^l \mathbf{H}^{l(t)} \right) \right), \tag{5}$$

where $B_{V,\text{self}}^l : \mathbb{R}^h \to \mathbb{R}^d$ and $B_V^l : \mathbb{R}^d \to \mathbb{R}^d$; the latter operates on each l-th head representation $\mathbf{H}^{l(t)}$ separately, mantaining their independence. The attention weights

are obtained with a slight modification of Eq. (2), by defining

$$\begin{aligned} \beta^l_{i,\mathrm{self}} &= (\mathbf{b}^l)^\top \mathrm{LeakyReLU}(B^l_Q \tilde{\mathbf{h}}^{(t)}_i + B^l_{K,\mathrm{self}} \tilde{\mathbf{h}}^{(t)}_i) \\ \beta^l_i &= (\mathbf{b}^l)^\top \mathrm{LeakyReLU}(B^l_Q \tilde{\mathbf{h}}^{(t)}_i + B^l_K \mathbf{H}^{l(t)} + B^l_E \mathbf{E}_i); \end{aligned} \tag{6}$$

these are then normalized using Softmax, as defined in Eq. (3), to obtain the final attention weights $\hat{\beta}^l_{i,\mathrm{self}}$ and $\hat{\beta}^l_i$. A Multi-layered Perceptron (MLP) mixes the contributions from different heads at the end of the broadcast phase, effectively integrating different classes of non-local interactions. Remarkably, RANGE requires the allocation of a fixed-sized vector, connected with a single edge to each node in the graph, enabling transfer of information across the system with a computational complexity that scales linearly with the number of nodes in the input graph. This is particularly advantageous when considering predictions on large systems, as it represents an improvement over standard FFT-based methods used for the treatment of long range interactions (e.g. Particle Mesh Ewald in the context of molecular dynamics), whose $N \log N$ scaling might represent a bottleneck during simulations of large molecules. While we considered a single master node in the description above, this design limits the amount of relevant global information that can be aggregated without loss, thereby constraining the scalability of the model. In the following section, we will address this limitation by introducing multiple master nodes, adapting the model to tasks where the number of nodes varies significantly across the dataset.

1.3 Spatial scalability

When several master nodes N_M with indices $I \in \{1 \dots N_M\}$ are employed, each one is initialized with a different embedding $\mathbf{H}^{(0)}_I$, and Eqs. (1) and (2) become, respectively,

$$\mathbf{H}^{(t)}_I = \sigma\left(\Vert^L_{l=1} \sum_i \alpha^l_{iI} A^l_V \mathbf{h}_i \right) \tag{7}$$

and

$$\alpha^l_{iI} = (\mathbf{a}^l)^\top \mathrm{LeakyReLU}(A^l_Q \mathbf{H}^{(t-1)}_I + A^l_K \mathbf{h}_i + A^l_E \mathbf{E}_{iI}). \tag{8}$$

In this context, the edge features $\mathbf{E}_{iI}$ can be master node-dependent but, in order to maximize parameter sharing without sacrificing performances, the same edge features are allocated for all master nodes. Similarly, the broadcast phase can be generalized to the case of multiple master nodes. Each d-dimensional portion of the output vector $\mathbf{h}^{(t+1)}_i$ can select from multiple global representations, and Eq. (5) and the second of Eq. (6) become, respectively,

$$\mathbf{h}^{(t+1)}_i = \mathrm{MLP}\left(\Vert^L_{l=1} \left(\hat{\beta}^l_{i,\mathrm{self}} B^l_{V,\mathrm{self}} \tilde{\mathbf{h}}^{(t)}_i + \sum_I \hat{\beta}^l_{iI} B^l_V \mathbf{H}^{l(t)}_I \right) \right) \tag{9}$$

and

$$\beta^l_{iI} = (\mathbf{b}^l)^\top \mathrm{LeakyReLU}(B^l_Q \tilde{\mathbf{h}}^{(t)}_i + B^l_K \mathbf{H}^{l(t)}_I + B^l_E \mathbf{E}_{iI}). \tag{10}$$

After normalizing, a regularization parameter

$$\lambda_I \in \begin{cases} \{1\} & \text{if } I = 1 \\ [0,1) & \text{if } I > 1, \end{cases} \tag{11}$$

biased on the system size, rescales the contribution from each master node during broadcast by:

$$\Lambda_I(n) = \lambda_I^{\gamma(n)} \tag{12}$$

$$\gamma(n) = (1 + a_I)|\max[0, (1-n)] + \tanh(b_I)\min[1, n]|. \tag{13}$$

Here, a_I and b_I are positive trainable parameters, and $n = (N - N_{\min})/(N_{\max} - N_{\min})$ is the normalized number of nodes in the graph, with $N_{\min}$ and $N_{\max}$ being the minimum and maximum number of nodes present in the dataset during training, respectively. While the scalar λ_1 is designed always to ensure at least one fully activated master node, the intensity of all the $\lambda_{I\neq 1}$ is controlled by the factor $\gamma(n)$ as a function of the system size n. Intuitively, $\gamma(n)$ should a) decrease with n, following the intuition that larger molecules need larger capacity per head, and b) always be greater than zero. Given these requirements, we opted for the parametric function in Eq. (13), enforcing $\gamma(n) > 1$ for small molecules and $\gamma(n) < 1$ for large molecules, with the values a_I and b_I controlling this behavior. Finally, the broadcast attention weights are rescaled as follows:

$$\hat{\beta}^l_{iI} \leftarrow \Lambda_I(n)\hat{\beta}^l_{iI} \qquad \text{for } I \in \{1 \dots N_M\}. \tag{14}$$

Approaches as the one delineated in Eq. (14), which aim at regularizing the overall usage of a given node in the trained model, are theoretically motivated [3] and have been proven effective in real word scenarios [4].

1.4 Application to equivariant models

Typically, SE(3)-equivariant MLFFs are designed considering 1) an invariant features representation, and 2) a set of high-order equivariant features; a mixing step is often implemented to exchange information between the two representations [5–8]. The RANGE aggregation and broadcast procedures, as defined in Eq. (1) and Eq. (5), cannot be directly applied to SE(3)-equivariant features due to the presence of nonlinear transformations. In agreement to other designs [9, 10], we transfer long-range information via the invariant features and possibly propagate it to the equivariant embeddings via the mixing step in the baseline model. While it is possible to explicitly incorporate higher-order equivariant features in the aggregation-broadcast scheme, this design choice maximizes computational efficiency and enables modularity in RANGE.

Supplementary Note 2: Datasets

The NaCl [11], AuMgO [11], and Biodimers [12, 13] datasets were calculated at DFT level of theory, using either the PBE or the HSE06 exchange-correlation functional. The QM7-X [14], AQM [15], and MD22 [16] atomic datasets were calculated at the

DFT level of theory, using either the PBE or PBE0 exchange-correlation functional and explicit treatment of van der Waals interactions via many-body dispersion (MBD) [17–20].

2.1 NaCl

The dataset contains 5 000 structures of $Na_9Cl_8^+$ and $Na_8Cl_8^+$ clusters. The reference data used to produce the results shown in Fig. 2a of the main text are taken from Rumiantsev et al. [21].

2.2 AuMgO

The dataset models an Au_2 dimer adsorbed on a periodic MgO surface, with and without an Al dopant located beneath the surface layer. The training structures include both *wetting* (flat) and *non-wetting* (upright) adsorption geometries on doped and undoped surfaces, for a total of 5 000 structures, while the testing configurations only contains *non-wetting* configurations. The data used to produce the results shown in Fig. 2b of the main text are taken from Rumiantsev et al. [21].

2.3 Biodimers

The dataset contains minimum energy organic side-chain dimers, including small molecules such as ethanol and acetamide. Dimers are classified by electronic distribution: polar, apolar, or charged. For each dimer, the intermolecular distance is systematically increased by up to 15 Å, resulting in 29 783 configurations. Following Rumiantsev et al. [21], samples with intermolecular distance exceeding the equilibrium distance by more than 4 Å (13 743 structures) form the test set; the remaining 16 040 structures constitute the training set. The dataset spans a wide variety of interaction regimes, from electrostatic- to dispersion-driven, providing a challenging test both at short- and long-range.

2.4 QM7-X

The QM7-X dataset comprises 42 physicochemical properties calculated for $\sim$4.2 millions equilibrium and non-equilibrium structures of organic molecules with up to 23 atoms. These cover the set of elements that is the most predominant in biomolecules, that is H, C, N, O, S, Cl. To better represent the effect of long-range interactions, a subset of QM7-X encompassing structures with more than 20 atoms was selected to train and validate the different models. The reduced dataset contains approximately 200 000 different structures, with 99% of all pairwise distances below 7 Å and an average of 3.4 ± 1.3 Å.

2.5 AQM

The Aquamarine dataset contains over 40 global and local physicochemical properties of $\sim$60 000 low- and high-energy conformers of 1 653 molecules with up to 92 atoms, both in gas phase and implicit water [15]. In our tests, we only considered the gas

phase version of the dataset and we further filtered out all structures with less than 30 atoms. This selection led to ∼52 000 structures with a mean pairwise distance between atoms of 6 ± 3 Å. Approximately 65% of all pairwise distances are below 7 Å, 83% are below 9 Å and 95% are below 12 Å.

2.6 MD22

We selected multiple systems from the MD22 dataset to cover a diverse range of molecular sizes and interaction types; specifically, the docosahexaenoic acid (*DHA*), the buckyball catcher, and the double-walled carbon nanotube.

2.6.1 DHA

The MD22 dataset contains ∼70 000 frames from molecular dynamics simulations of DHA, a biologically relevant lipid composed of 56 atoms. The mean pairwise distance between atoms in each frame is 6 ± 3 Å, with 63% of pairs below 7 Å, 81% below 9 Å, and 94% below 12 Å.

2.6.2 Buckyball Catcher

The Buckyball catcher dataset consists of ∼6 000 frames of the $C_{120}H_{28}$ supramolecule. The mean pairwise distance between the atoms in the system is 7 ± 3 Å, with 43% of pairs below 7 Å, 64% below 9 Å, and 90% below 12 Å.

2.6.3 Double-Walled Nanotube

The double-walled nanotube dataset contains ∼5 000 frames of the large $C_{326}H_{44}$ supramolecule. The mean pairwise distance between the atoms in the system is 11 ± 6 Å, with 25% of pairs below 7 Å, 41% below 9 Å, and 64% below 12 Å.

Supplementary Note 3: Model training

All the models where trained using the combined force and energy loss:

$$\mathcal{L} = \alpha \sum_{i=1}^{N} \frac{1}{n_i} |E_i - E(\mathbf{X_i}; \theta)|^2 + \sum_{i=1}^{N} |\mathbf{F}_i + \nabla E(\mathbf{X_i}; \theta)|^2. \tag{15}$$

Here, N is the number of molecules, n_i is the number of atoms in the i-th molecule, E_i and $\mathbf{F}_i$ are the potential energy and forces acting on the i-th molecule. $E(\mathbf{X_i}; \theta)$ and $\nabla E(\mathbf{X_i}; \theta)$ are the energy and forces predicted by the model, which depend on the network parameters θ. Finally, α is a scalar value that controls the relative numerical weight between force and energy contributions. A term that acts specifically on the parameters that regulate the activation of multiple master nodes is introduced in the loss function as

$$\mathcal{L}_{\text{reg}} = \sum_{I} \delta |\lambda_I + a_I + b_I|, \tag{16}$$

where the scalar δ was set to 2.0 during all the trainings. All models were trained for 200 epochs with the exception of the MACE architecture, for which training was extended to 600 epochs, and the training on the MD22 dataset, which was performed for 500 epochs. The AdamW [22] optimizer was used in all training, with an initial learning rate of 0.0001 and a weight decay of 0.01 (0.001 and 5×10^{-7} for MACE). For the first 125 epochs (400 for MACE), α was set to ~ 0.01 times the average number of atoms in the dataset, and subsequently increased by a factor 10. A linear scheduler was used with a learning rate decay of 0.8 and learning rate step size of 19 (57 for MACE) to optimize the model parameters. The value of the regularization parameter λ_I was set to 6, and the parameters a_I and b_I were set to 8. To scale different parameter groups with different step sizes, we employed a custom implementation of the standard *LinearLR* class in the PyTorch library [23]. Model hyperparameters are reported in the Supplementary Tables 1 and 2. All models, with the exception of Neural P^{3}M for which the original codebase was used, were trained using the mlcg package [24].

Supplementary Table 1: Training hyperparameters. Neural network hyperparameters used for all baseline models and their RANGE counterparts.

	Training setup
Hidden channels (h)	144
Master nodes hidden channels (h')	432
Number of Filters (L)	144
Interaction Blocks (T)	3-2
Activation	Tanh-(MACE & SO3krates: SilU)
Cutoff function	CosineCutoff
Distance Expansion Basis	Gaussian RBF [25]-(MACE: Bessel)
Master node RBF dimension	7
Output Network	MLP, 2 layers, [128,64] features
Output Prediction	energy, forces
Attention heads	8
MACE `max_ell`	3
MACE `correlation`	3
MACE `hidden_irreps`	144x0e + 144x1o + 144x2e
MACE `distance_transform`	Agnesi
SO3krates `degrees`	[1, 2, 3]
SO3krates `num_heads`	4

3.1 Number of virtual nodes versus embedding dimension

A comparative numerical study has been conducted to assess whether multiple virtual nodes (VNs) perform better than a single one with larger embedding size. For this analysis, we have used PaiNN as a baseline to balance model complexity and resource usage. In Supplementary Fig. 1, mean absolute error (MAE) and root mean squared error (RMSE) are shown for the AQM test set. We note a significant improvement in performances when adding multiple VNs, each with independent initial embedding but

Supplementary Table 2: Radial basis expansion. Dimension of the radial basis expansion used in all baseline models and their RANGE counterparts for different cut-off radii, with the exception of MACE-based models, where 8 basis functions are used for both 5 Å and 7 Å cutoffs.

Radius (Å)	Number of RBF
4.0	27
5.0	32
7.0	45
9.0	58
12.0	77

shared weights. This behavior justifies our choice over merely increasing the hidden dimension of a single VN. The different VNs are regularized according to the procedure presented in the main text, imposing a size-aware model behavior. The error bar is calculated from the standard deviation over five realizations with different seed of the stochastic optimization algorithm.

3.2 Performance evaluation

To ensure accurate and reliable evaluation of the metrics, inference time and GPU memory usage were evaluated on dedicated GPUs in an isolated environment to avoid slowdowns due to thermal and power fluctuations. Temperature and power were monitored throughout each run as indicators of experimental stability. For each model, both metrics were obtained during inference after an initial warm-up phase.
The reported values were obtained considering the average and standard deviation over a minimum of five independent runs. The number of runs was increased to 50 if the relative error resulted grater than 10%.
For Neural P^3M, to accurately reflect real deployment conditions, the inference time reported in this work includes the grid construction over the molecular frames. While this operation is often excluded during training because it is performed as a preprocessing step, at inference time it is necessary to recalculate the grid at each step. We included its calculation to ensure a fair comparison across methods.
The batch size used for computing the metrics on the AQM dataset was 64 for MACE-based models, 128 for PaiNN- and SO3krates-based models, and 512 for SchNet-based models. For MD22, batch sizes of 64, 16, and 2 were used for the DHA, buckyball catcher, and double-walled nanotube dataset, respectively.
In the scaling tests reported in Supplementary Fig. 2, the inference time is measured on artificial systems generated by uniformly distributing an increasing number of atoms within a cubic box, whose side length was chosen to maintain an average

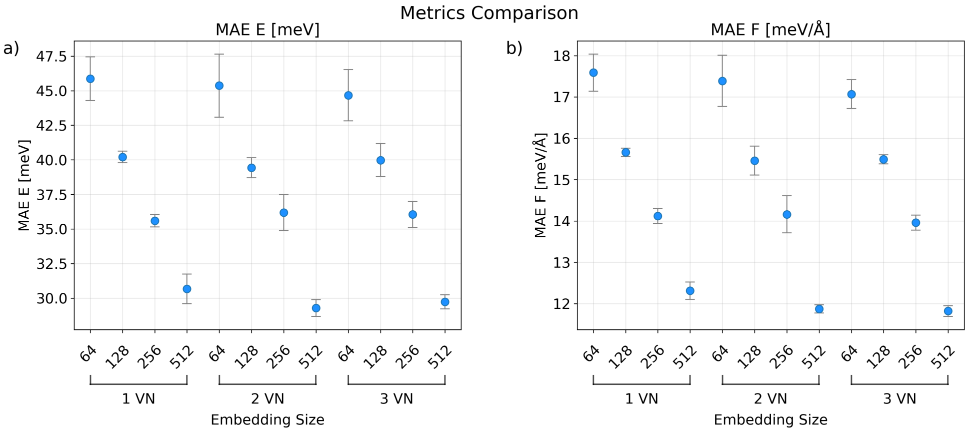

Supplementary Figure 1: Metrics against number of VN and respective hidden dimension. The MAE of energy a) and forces b) for PaiNN+RANGE models trained on the AQM dataset are plotted against the hidden dimension for different virtual nodes. All the reported values are averaged over 4 models independently trained with different dataset seeds. Error bars correspond to standard deviation. Source data are provided as a Source Data file.

density of 0.1 Å^{-3}.

Supplementary Note 4: Simulation details

All-atom simulations were conducted using a PaiNN+RANGE model with a baseline cutoff of 5.0 Å, 3 master nodes, and 8 attention heads for stability analysis. Each simulation was run for 16 ns using a Langevin integrator at 300 K, with a timestep of 1 fs. To gather robust statistics on the conformational space exploration by each model, 10, 5 and 2 parallel simulations were performed for DHA, buckyball catcher and double-walled nanotube, respectively. All simulations remained stable for their entire duration, confirming that the model can support long, stable molecular dynamics runs. The scaling performance during molecular dynamic simulations is reported in Supplementary Fig. 4 for all the systems.

Supplementary Note 5: Interpretation and singular value decomposition analysis

For each configuration in the validation set V_{data} of DHA, buckyball catcher, and double-walled nanotube, two N dimensional vectors, containing aggregation and broadcast weights of the master node with $\lambda_1 = 1$ during the last interaction block,

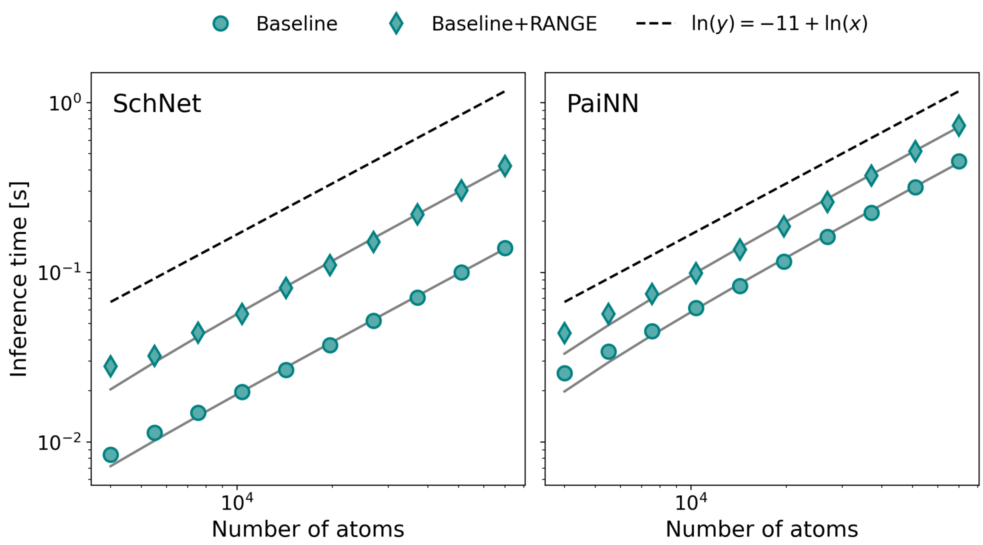

Supplementary Figure 2: Inference time scaling. The inference time of RANGE scales linearly with the number of atoms. Test systems in the 4 000-70 000 atoms range were generated by randomly placing atoms within a cubic box to achieve an atomic density of 0.1 $Å^{-3}$. Linear fits for each model are shown as solid gray lines to highlight the linear dependence on the number of atoms. Source data are provided as a Source Data file.

are stored as matrix rows to analyze the attention patterns of the RANGE model. The two matrices of size $|V_{\mathrm{data}}| \times N$ are decomposed in singular values for every attention head separately. Supplementary Figs. 5, 6 and 7 show the results for aggregation and broadcast of each of the aforementioned molecules respectively. Singular values within each matrix are normalized with respect to their maximum, highlighted in red. A single, dominant pattern associated to an N-dimensional principal component emerges, and its coefficients can be mapped onto the molecular graph with a color index, as shown in Fig. 5 of the main text.

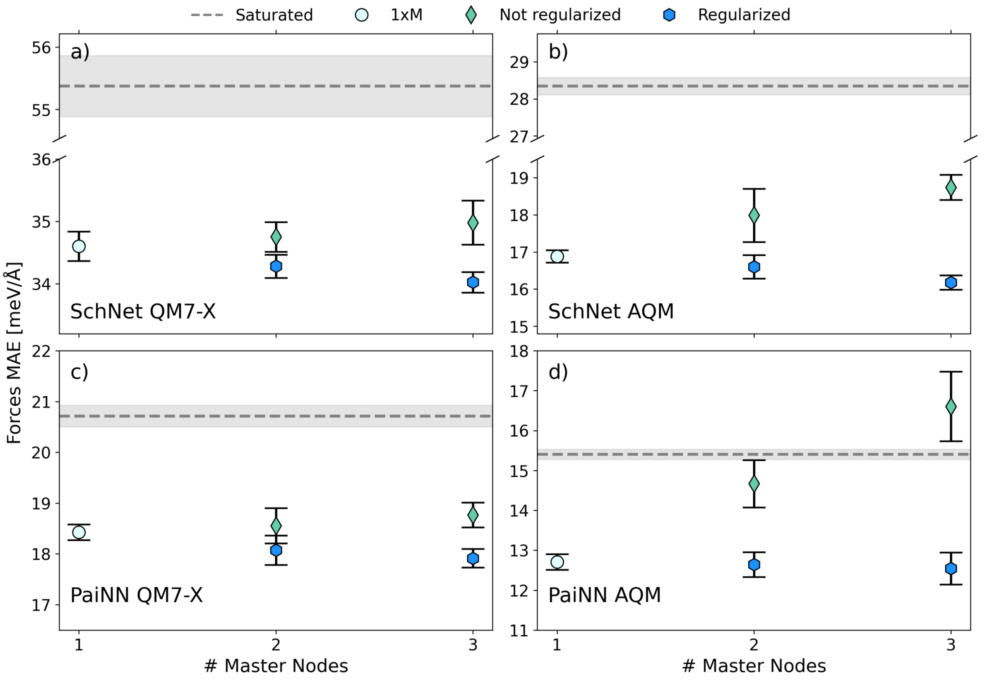

Supplementary Figure 3: MAE of the regularized and non-regularized RANGE model. Forces MAE of the regularized and non-regularized RANGE models with different number of master nodes are reported for the a) SchNet on the QM7-X, b) SchNet on the AQM, c) PaiNN on the QM7-X and d) PaiNN on the AQM datasets. The gray line represents the lowest MAE achieved by the baseline model upon increasing the message-passing cutoff. All the reported values are averaged over 4 models independently trained with different dataset seeds. Error bars correspond to standard deviation. Source data are provided as a Source Data file.

Supplementary Table 3: Accuracy and training on NaCl, AuMgO and biodimers datasets. Energy and forces MAE for the test set of NaCl, AuMgO and biodimers datasets are reported for different baseline and RANGE-augmented models. The best results are in bold lettering. Source data are provided as a Source Data file.

	Model	**NaCl** MAE energy [meV/atom]	**NaCl** MAE forces [meV/Å]	**AuMgO** MAE energy [meV/atom]	**AuMgO** MAE forces [meV/Å]	**Biodimers** MAE energy [meV/atom]	**Biodimers** MAE forces [meV/Å]
Baseline	SchNet	0.052	31.6	0.56	46.6	1.24	6.2
	PaiNN	0.050	27.6	0.57	37.4	1.08	5.8
	SO3krates	0.049	24.8	0.62	40.9	0.77	5.8
	MACE	0.049	25.2	0.0027	14.7	1.54	7.0
+RANGE	SchNet	0.0013	8.5	0.0030	19.9	0.14	4.3
	PaiNN	0.0011	7.0	0.0032	15.9	**0.10**	4.3
	SO3krates	**0.0007**	**5.6**	0.0075	13.3	0.14	**3.4**
	MACE	0.0077	6.3	**0.0007**	**10.5**	0.69	5.2

Supplementary Table 4: Different RANGE models on QM7-X. Energy and forces MAE are reported for different SchNet and PaiNN plus RANGE model with varying number of master nodes M (1, 2, and 3). Non regularized RANGE models are indicated as RANGE-NR. All the reported values are averaged over 4 models independently trained with different dataset seeds. The best results are in bold lettering. Source data are provided as a Source Data file.

	Model	MAE energy [meV/atom]	MAE forces [meV/Å]
SchNet	Baseline 4 Å	0.22 ± 0.02	60.6 ± 0.3
	Baseline 5 Å	0.19 ± 0.02	58.2 ± 0.2
	Baseline 7 Å	0.17 ± 0.02	56.4 ± 0.5
	Baseline 9 Å	0.17 ± 0.02	55.4 ± 0.5
	RANGE 4 Å (1xM)	$\mathbf{0.068} \pm 0.012$	34.6 ± 0.2
	RANGE 4 Å (2xM)	$\mathbf{0.063} \pm 0.007$	$\mathbf{34.3} \pm 0.2$
	RANGE 4 Å (3xM)	$\mathbf{0.067} \pm 0.008$	$\mathbf{34.0} \pm 0.2$
	RANGE-NR 4 Å (2xM)	$\mathbf{0.068} \pm 0.008$	34.8 ± 0.2
	RANGE-NR 4 Å (3xM)	$\mathbf{0.071} \pm 0.010$	35.0 ± 0.4
PaiNN	Baseline 4 Å	0.034 ± 0.006	22.6 ± 0.2
	Baseline 5 Å	0.030 ± 0.006	21.1 ± 0.1
	Baseline 7 Å	0.028 ± 0.008	20.7 ± 0.2
	Baseline 9 Å	0.028 ± 0.005	20.6 ± 0.1
	RANGE 4 Å (1xM)	$\mathbf{0.024} \pm 0.005$	18.4 ± 0.2
	RANGE 4 Å (2xM)	$\mathbf{0.021} \pm 0.004$	$\mathbf{18.1} \pm 0.3$
	RANGE 4 Å (3xM)	$\mathbf{0.021} \pm 0.006$	$\mathbf{17.9} \pm 0.2$
	RANGE-NR 4 Å (2xM)	$\mathbf{0.024} \pm 0.006$	18.6 ± 0.3
	RANGE-NR 4 Å (3xM)	$\mathbf{0.022} \pm 0.004$	18.8 ± 0.2

Supplementary Table 5: Different RANGE models on AQM datasets. Energy and forces MAE are reported for different SchNet and PaiNN plus RANGE model with varying number of master nodes M (1, 2, and 3). Non regularized RANGE models are indicated as RANGE-NR. All the reported values are averaged over 4 models independently trained with different dataset seeds. The best results are in bold lettering. Source data are provided as a Source Data file.

	Model	MAE energy [meV/atom]	MAE forces [meV/Å]
SchNet	Baseline 5 Å	0.222 ± 0.014	29.9 ± 0.2
	RANGE 5 Å (1xM)	$\mathbf{0.049} \pm 0.003$	16.9 ± 0.2
	RANGE 5 Å (2xM)	$\mathbf{0.047} \pm 0.001$	$\mathbf{16.6} \pm 0.3$
	RANGE 5 Å (3xM)	$\mathbf{0.046} \pm 0.003$	$\mathbf{16.2} \pm 0.2$
	RANGE-NR 5 Å (2xM)	0.056 ± 0.004	18.0 ± 0.7
	RANGE-NR 5 Å (3xM)	0.064 ± 0.002	18.7 ± 0.3
PaiNN	Baseline 5 Å	0.066 ± 0.005	16.2 ± 0.2
	RANGE 5 Å (1xM)	$\mathbf{0.035} \pm 0.002$	$\mathbf{12.7} \pm 0.2$
	RANGE 5 Å (2xM)	$\mathbf{0.035} \pm 0.003$	$\mathbf{12.6} \pm 0.3$
	RANGE 5 Å (3xM)	$\mathbf{0.037} \pm 0.003$	$\mathbf{12.5} \pm 0.4$
	RANGE-NR 5 Å (2xM)	0.049 ± 0.006	14.7 ± 0.6
	RANGE-NR 5 Å (3xM)	0.063 ± 0.007	16.6 ± 0.9

Supplementary Table 6: Accuracy, inference time and memory peak on the AQM dataset. Accuracy, inference time and memory peak are reported for different SchNet, PaiNN, SO3krates and MACE models, and their RANGE-corrected variants. All the reported MAE values are averaged on 4 models independently trained with different dataset seeds. The best results are in bold lettering. Source data are provided as a Source Data file.

	Model	MAE energy [meV/atom]	MAE forces [meV/Å]	Inference time [s]	Memory Peak [MB]
SchNet	Baseline 5 Å	0.222 ± 0.014	29.9 ± 0.2	0.446 ± 0.009	8074 ± 2
	Baseline 7 Å	0.176 ± 0.009	28.5 ± 0.3	0.552 ± 0.008	13137 ± 2
	Baseline 9 Å	0.166 ± 0.003	28.4 ± 0.3	0.637 ± 0.008	17012 ± 1
	Baseline 12 Å	0.150 ± 0.005	28.3 ± 0.2	0.714 ± 0.007	20187 ± 1
	RANGE 5 Å	**0.046** ± 0.003	**16.2** ± 0.2	0.690 ± 0.007	13089 ± 4
	RANGE 7 Å	**0.043** ± 0.001	**16.0** ± 0.3	0.801 ± 0.010	17858 ± 4
	RANGE 9 Å	**0.042** ± 0.001	**16.0** ± 0.04	0.880 ± 0.007	21521 ± 5
	RANGE 12 Å	**0.045** ± 0.002	16.4 ± 0.2	0.958 ± 0.009	24548 ± 4
PaiNN	Baseline 5 Å	0.066 ± 0.005	16.2 ± 0.2	1.01 ± 0.01	7010 ± 2
	Baseline 7 Å	0.052 ± 0.001	15.4 ± 0.1	1.38 ± 0.02	11333 ± 3
	Baseline 9 Å	0.049 ± 0.002	15.4 ± 0.1	1.79 ± 0.03	14439 ± 3
	Baseline 12 Å	0.045 ± 0.002	15.5 ± 0.1	1.99 ± 0.02	16749 ± 3
	RANGE 5 Å	0.036 ± 0.003	12.5 ± 0.4	1.4 ± 0.02	8455 ± 4
	RANGE 7 Å	**0.027** ± 0.001	**11.6** ± 0.3	1.79 ± 0.08	12780 ± 4
	RANGE 9 Å	**0.026** ± 0.001	**11.5** ± 0.2	2.19 ± 0.02	15892 ± 5
	RANGE 12 Å	**0.026** ± 0.001	**11.7** ± 0.3	2.38 ± 0.02	18206 ± 5
SO3krates	Baseline 5 Å	0.061 ± 0.003	16.9 ± 0.1	1.17 ± 0.03	7505 ± 5
	Baseline 7 Å	0.059 ± 0.003	17.0 ± 0.2	1.54 ± 0.02	12124 ± 5
	Baseline 9 Å	0.056 ± 0.001	16.9 ± 0.3	1.80 ± 0.08	15628 ± 4
	Baseline 12 Å	0.057 ± 0.003	17.6 ± 0.6	2.17 ± 0.02	18167 ± 6
	RANGE 5 Å	**0.035** ± 0.003	**12.8** ± 0.3	1.58 ± 0.04	9077 ± 13
	RANGE 7 Å	**0.032** ± 0.002	**12.5** ± 0.3	1.93 ± 0.04	13684 ± 6
	RANGE 9 Å	**0.031** ± 0.002	**12.5** ± 0.3	2.15 ± 0.03	17202 ± 6
	RANGE 12 Å	**0.032** ± 0.002	13.0 ± 0.1	2.53 ± 0.07	19734 ± 6
MACE	Baseline 5 Å	0.133 ± 0.029	13.8 ± 0.3	2.25 ± 0.04	13193 ± 0
	Baseline 7 Å	0.209 ± 0.049	17.5 ± 0.3	2.72 ± 0.02	21298 ± 1
	Baseline 9 Å	0.308 ± 0.046	21.7 ± 0.4	3.07 ± 0.02	27065 ± 1
	Baseline 12 Å	0.545 ± 0.067	30.8 ± 1.3	3.34 ± 0.09	31211 ± 0
	RANGE 5 Å	**0.057** ± 0.008	**10.4** ± 0.3	2.48 ± 0.10	13663 ± 1
	RANGE 7 Å	0.068 ± 0.006	12.8 ± 0.3	2.93 ± 0.02	21769 ± 0
	RANGE 9 Å	0.103 ± 0.014	15.9 ± 0.9	3.28 ± 0.02	27539 ± 2
	RANGE 12 Å	0.202 ± 0.017	22.7 ± 1.4	3.55 ± 0.05	31683 ± 1

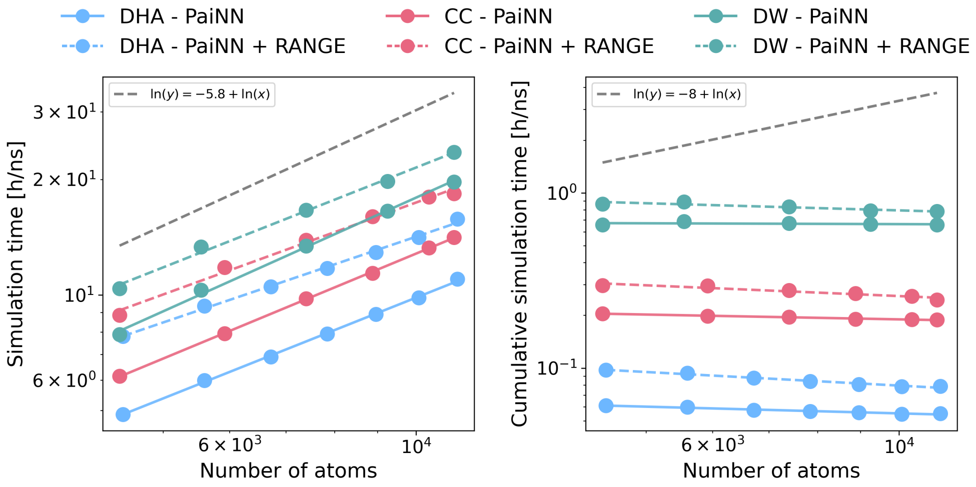

Supplementary Figure 4: Scaling performance of molecular dynamics simulations. Left: Simulation time (hours per nanosecond) as a function of the total number of atoms in a batch of simulations run in parallel. Right: Cumulative simulation time, obtained by multiplying the simulation time by the number of structures in the batch. The total number of atoms in both panels refers to the number of atoms per molecule multiplied by the total number of parallel configurations simulated. Source data are provided as a Source Data file.

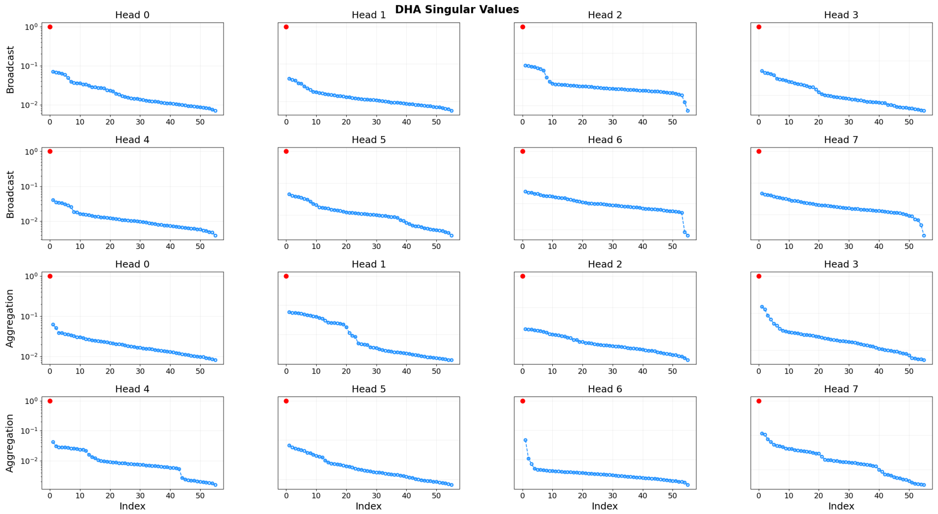

Supplementary Figure 5: Singular value decomposition (SVD) of aggregation and broadcast weights. The SVD analysis is performed for DHA on the master node with $\lambda_1 = 1$. Its principal component, corresponding to the largest value, is marked in red. Source data are provided as a Source Data file.

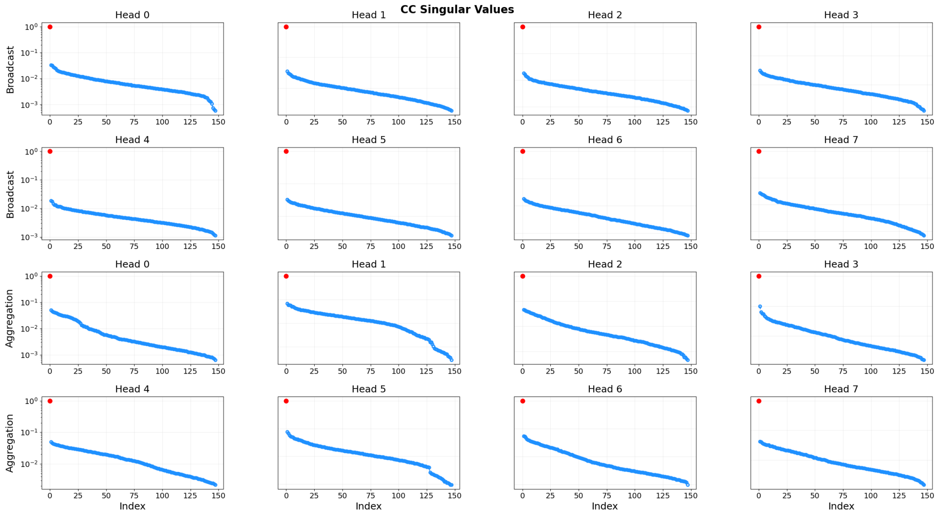

Supplementary Figure 6: Singular value decomposition (SVD) of aggregation and broadcast weights. The SVD analysis is performed for the buckyball catcher on the master node with $\lambda_1 = 1$. Its principal component, corresponding to the largest value, is marked in red. Source data are provided as a Source Data file.

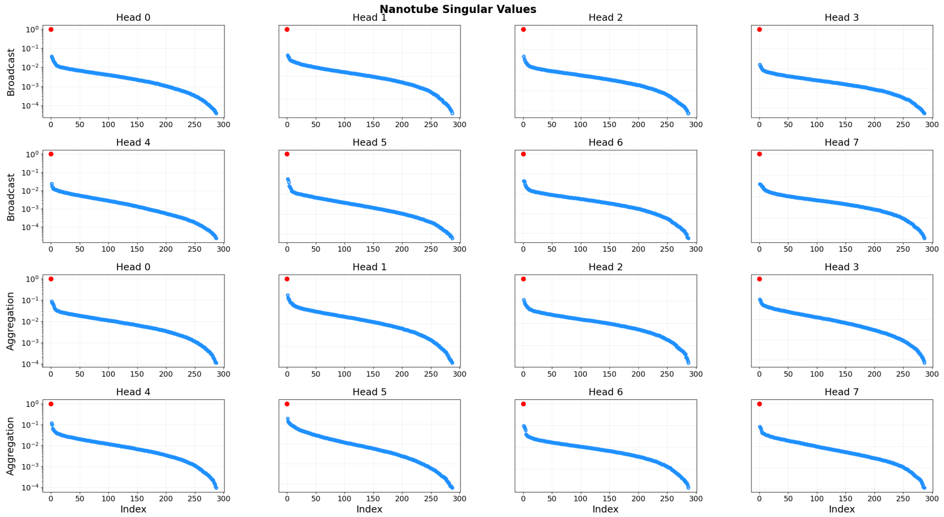

Supplementary Figure 7: Singular value decomposition (SVD) of aggregation and broadcast weights. The SVD analysis is performed for the double-walled nanotube on the master node with $\lambda_1 = 1$. Its principal component, corresponding to the largest value, is marked in red. Source data are provided as a Source Data file.

References

[1] Veličković, P. *et al.* Graph attention networks. *arXiv preprint arXiv:1710.10903* (2017).

[2] Brody, S., Alon, U. & Yahav, E. How attentive are graph attention networks? *arXiv preprint arXiv:2105.14491* (2021).

[3] Giambagli, L., Buffoni, L., Chicchi, L. & Fanelli, D. How a student becomes a teacher: learning and forgetting through spectral methods. *Adv. Neural Inf. Process.* **36**, 60291–60306 (2023).

[4] Liu, Z. *et al.* Learning efficient convolutional networks through network slimming. *Proceedings of the IEEE international conference on computer vision* 2736–2744 (2017).

[5] Satorras, V. G., Hoogeboom, E. & Welling, M. E(n) equivariant graph neural networks. *International conference on machine learning* 9323–9332 (2021).

[6] Schütt, K. T., Unke, O. & Gastegger, M. Equivariant message passing for the prediction of tensorial properties and molecular spectra. *International Conference on Machine Learning* 9377–9388 (2021).

[7] Batatia, I., Kovacs, D. P., Simm, G., Ortner, C. & Csányi, G. Mace: Higher order equivariant message passing neural networks for fast and accurate force fields. *Adv. Neural Inf. Process.* **35**, 11423–11436 (2022).

[8] Fu, X. *et al.* Forces are not enough: Benchmark and critical evaluation for machine learning force fields with molecular simulations. *arXiv preprint arXiv:2210.07237* (2022).

[9] Kosmala, A., Gasteiger, J., Gao, N. & Günnemann, S. Ewald-based long-range message passing for molecular graphs. *International Conference on Machine Learning* 17544–17563 (2023).

[10] Wang, Y. *et al.* Neural p^3m: A long-range interaction modeling enhancer for geometric gnns. *arXiv preprint arXiv:2409.17622* (2024).

[11] Ko, T. W., Finkler, J. A., Goedecker, S. & Behler, J. A fourth-generation high-dimensional neural network potential with accurate electrostatics including non-local charge transfer. *Nat. Commun.* **12**, 398 (2021).

[12] Burns, L. A. *et al.* The biofragment database (bfdb): An open-data platform for computational chemistry analysis of noncovalent interactions. *J. Chem. Phys.* **147** (2017).

[13] Huguenin-Dumittan, K. K., Loche, P., Haoran, N. & Ceriotti, M. Physics-inspired equivariant descriptors of nonbonded interactions. *J. Phys. Chem. Lett.* **14**, 9612–9618 (2023).

[14] Hoja, J. *et al.* Qm7-x, a comprehensive dataset of quantum-mechanical properties spanning the chemical space of small organic molecules. *Sci. Data* **8**, 43 (2021).

[15] Medrano Sandonas, L. *et al.* Dataset for quantum-mechanical exploration of conformers and solvent effects in large drug-like molecules. *Sci. Data* **11**, 742 (2024).

[16] Chmiela, S. *et al.* Accurate global machine learning force fields for molecules with hundreds of atoms. *Sci. Adv.* **9**, eadf0873 (2023).

[17] Tkatchenko, A., DiStasio Jr, R. A., Car, R. & Scheffler, M. Accurate and efficient method for many-body van der waals interactions. *Phys. Rev. Lett.* **108**, 236402 (2012).

[18] Ambrosetti, A., Reilly, A. M., DiStasio, R. A. & Tkatchenko, A. Long-range correlation energy calculated from coupled atomic response functions. *J. Chem. Phys.* **140** (2014).

[19] Stöhr, M., Michelitsch, G. S., Tully, J. C., Reuter, K. & Maurer, R. J. Communication: Charge-population based dispersion interactions for molecules and materials. *J. Chem. Phys.* **144** (2016).

[20] Mortazavi, M., Brandenburg, J. G., Maurer, R. J. & Tkatchenko, A. Structure and stability of molecular crystals with many-body dispersion-inclusive density functional tight binding. *J. Phys. Chem. Lett.* **9**, 399–405 (2018).

[21] Rumiantsev, E., Langer, M. F., Sodjargal, T.-E., Ceriotti, M. & Loche, P. Learning long-range representations with equivariant messages. *arXiv preprint arXiv:2507.19382* (2025).

[22] Loshchilov, I. & Hutter, F. Decoupled weight decay regularization. *arXiv preprint arXiv:1711.05101* (2017).

[23] Paszke, A. *et al.* Pytorch: An imperative style, high-performance deep learning library. *Adv. Neural Inf. Process.* **32** (2019).

[24] Charron, N. E. *et al.* Navigating protein landscapes with a machine-learned transferable coarse-grained model. *Nat. Chem.* 1–9 (2025).

[25] Schütt, K. T. *et al.* Schnet: A continuous-filter convolutional neural network for modeling quantum interactions. *Adv. Neural Inf. Process.* **30**, 992–1002 (2017).
